# Methods of Feces Disposal and Fly Prevention in the French Army

**Published:** 1918-10

**Authors:** Octave Morod


### FIELD SANITATION

**Methods of Feces Disposal and Fly Prevention in the French Army.** By Medecin-Major Octave MONOD.

The speaker said in part:

Of the problems which the sanitary authorities in an army have to solve, two are particularly important: the destruction of fecal matter, and the fight against the fly.

The speaker said that he would treat the subject simply from the practical point of view. His remarks, he said, were based largely upon circulars published since the beginning of the war by the Service de Santé and the General Headquarters.

*The Destruction of Fecal Matter.* This may be accomplished either by incineration or by burial. The former procedure is the usual one in the English Army; the second, on the contrary, is almost exclusively employed in the French Army.

Burning has the advantage of destroying rapidly and completely all organisms of a dangerous character present in the fecal matter.

The simple procedure of burial should be accompanied with certain precautions (set forth below).

When the troops are at rest, whether in special camps or in inhabited villages, it is indispensable to have available a considerable number of pits carefully distributed so as to prevent the necessity for men to defecate at large.

In the villages and in places where pits may not be dug for fear of contaminating the veins of water underlying the surface of the soil, recourse must be had to metal receptacles. A ditch for the disposal of the contents of the receptacles may then be dug at a point where contamination of the water supply need not be feared.

Every day this pit should be sprinkled with disinfectants, such as crude oil, cresol, and quick lime, and covered over with earth.

*The Fight against the Fly.* It is very evident that all precautions taken against fecal matter concern the fly problem as well. The fly is as dangerous as it is disagreeable and filthy. It is frequently a means of propagating disease. It may be suppressed in two ways: first, by preventing the hatching of the fly's eggs; and second, by the destruction of the fly itself after it is hatched.

Although there are many varieties of flies in France, the methods of combating them are not as numerous.

The house-fly lays its eggs on all kinds of organic matter in process of decomposition. Fresh horse manure appears to be the most likely place of deposit. About 90 per cent of fly infestation may be traced to this source.

The eggs are layed on the surface of the manure, where the newly hatched remain until about the sixth day, when they migrate to the lower strata before complete development. The young flies are so delicate that they require the protection of a house, and if houses are sufficiently removed from the place of propagation they are not likely to become infested.

In order to prevent the hatching of the flies, the manure must be removed daily from the neighborhood of the troops. The manure should be burned whenever possible. In each district there should be an incinerating furnace. If, however, incineration is not possible, the dejecta must be buried in a deep and narrow ditch, which should be sprinkled daily with a 50 per cent solution of crude oil. When the ditch is three fourths filled, it should be completely covered with earth.

*Slaughter-House Disinfection.* The slaughter-house center ought to be infected likewise. The cattle pens should be sprinkled with iron sulphate (10 o/o).

The debris from the animals should be burned, or at least buried after being thoroughly sprinkled with iron sulphate powder and covered with tar to keep away the rats.

*Dead Bodies.* The removal of dead bodies from the field of battle is often a difficult and dangerous operation on account of exposure to bombardment and attack. The burial of bodies near the first lines is rarely attempted, as it is better to remove them to a less exposed region before attempting to inter them. This enables the workers to dig the pits more carefully and to carry out the proper sanitary measures.

Small camionette automobiles facilitate the search for and removal of the dead bodies. When they have been withdrawn to the rear, the bodies are placed in a pit of about one and a half meters in depth. They are then covered with a layer of quick lime or they are covered with a preparation of crude oil and cresol. When bodies are too badly mutilated or decomposed to permit of removal, they must, of course, be buried with whatever care is possible.

The bodies of animals, which of course cannot be removed, must also be buried without removal and as soon as possible.

Dead bodies near the trenches, which cannot be removed, must be covered with quick lime or sprinkled with the solution of crude oil and cresol. They may be effectively isolated by the construction of a wooden shield filled with lime and earth, or by a sufficient quantity of crude oil and coal tar.

*Protection against Winged Insects.* When all the precautions already mentioned are taken, the hatching of flies may be greatly reduced. These measures, however, meet with many obstacles which prevent a complete realization. Flies are especially dangerous by reason of their going and coming between the decaying organic matter and the food depots or cooking establishments. Every precaution is therefore necessary to protect the preparation and storing of food against this grave danger of infection.

The store houses in which fresh food is kept must be screened by a wire netting. The bread supplies and all others must be guarded in this fashion, and as the wire or gauze netting necessary for the purpose is easily transported, this precaution may be easily taken anywhere and at any time.

The speaker described a cabinet contrived by him to afford such protection. The walls were of cloth or gauze, kept out by the weight of the tray forming the base as the whole hangs from a hook in the ceiling.

As flies avoid dark places, it is well to avoid as far as possible the lighting of store houses by windows or other openings.

In spite of all precautions, flies succeed in penetrating the barriers. It is therefore necessary to consider the means of ridding the stores of flies. The means most frequently employed are as follows :

1. A solution of formol is placed in a plate (2 parts formol to 100 of water).

2. The following mixture may be used instead :

5 parts of formol.
25 parts of milk or butter milk.
60 parts of slightly sweetened water.

A few pieces of bread are floated in the solutions to attract the flies. The plates should not be placed in a draught, and their number should vary according to the size of the room.

3. A sticky tanglefoot may be prepared by melting :

72 parts of gum in 50 parts of castor oil.
12 parts of honey or of glucose are added.

Slender rods are dipped in this preparation and stuck up in receptacles filled with sand.

6. Another tanglefoot preparation is :

15 parts castor oil.
8 parts of resin.

The mixture is obtained by boiling. (This preparation is used in the British Army.)

When it is desirable to kill at once all the flies in a given place, sulphur or cresol fumes may be used.

1. Sulphur fumes may be produced by combustion of stick sulphur, allowing 30 grams for each cubic meter to be disinfected.

2. The amount of cresol to be vaporized is about 5 grams per cubic meter of the air space to be disinfected. This quantity of cresol is heated in a metal receptacle, so as to avoid the danger of conflagration. The fumes thus given off are very toxic for insects, but wholly inocuous for men. The vapor should be left in the chamber from four to five hours. It is often convenient to allow fumes to remain during the night.

The speaker briefly alluded to the admirable sanitary organization effected by Monsieur Godart, who made provision for a permanent body of hygienic officers in each Army Zone. A maximum of comfort and sanitary environment has in general been provided, so far as the conditions of war permit.

